# Lipid Emulsion Improves Functional Recovery in an Animal Model of Stroke

**DOI:** 10.3390/ijms21197373

**Published:** 2020-10-06

**Authors:** Motomasa Tanioka, Wyun Kon Park, Joohyun Park, Jong Eun Lee, Bae Hwan Lee

**Affiliations:** 1 Department of Physiology, Yonsei University College of Medicine, Seoul 03722, Korea; hpark@yuhs.ac; 2 Brain Korea 21 PLUS Project for Medical Science, Yonsei University College of Medicine, Seoul 03722, Korea; jhpark922@yuhs.ac (J.P.); jelee@yuhs.ac (J.E.L.); 3 Department of Anesthesiology and Pain Medicine, Anesthesia and Pain Research Institute, Yonsei University College of Medicine, Seoul 03722, Korea; wkp7ark@yuhs.ac; 4 Department of Anatomy, Yonsei University College of Medicine, Seoul 03722, Korea

**Keywords:** neuroprotection, stroke, ischemia, middle cerebral artery occlusion, reperfusion injury, lipid emulsion, excitotoxicity

## Abstract

Stroke is a life-threatening condition that leads to the death of many people around the world. Reperfusion injury after ischemic stroke is a recurrent problem associated with various surgical procedures that involve the removal of blockages in the brain arteries. Lipid emulsion was recently shown to attenuate ischemic reperfusion injury in the heart and to protect the brain from excitotoxicity. However, investigations on the protective mechanisms of lipid emulsion against ischemia in the brain are still lacking. This study aimed to determine the neuroprotective effects of lipid emulsion in an in vivo rat model of ischemic reperfusion injury through middle cerebral artery occlusion (MCAO). Under sodium pentobarbital anesthesia, rats were subjected to MCAO surgery and were administered with lipid emulsion through intra-arterial injection during reperfusion. The experimental animals were assessed for neurological deficit wherein the brains were extracted at 24 h after reperfusion for triphenyltetrazolium chloride staining, immunoblotting and qPCR. Neuroprotection was found to be dosage-dependent and the rats treated with 20% lipid emulsion had significantly decreased infarction volumes and lower Bederson scores. Phosphorylation of Akt and glycogen synthase kinase 3-β (GSK3-β) were increased in the 20% lipid-emulsion treated group. The Wnt-associated signals showed a marked increase with a concomitant decrease in signals of inflammatory markers in the group treated with 20% lipid emulsion. The protective effects of lipid emulsion and survival-related expression of genes such as Akt, GSK-3β, Wnt1 and β-catenin were reversed by the intra-peritoneal administration of XAV939 through the inhibition of the Wnt/β-catenin signaling pathway. These results suggest that lipid emulsion has neuroprotective effects against ischemic reperfusion injury in the brain through the modulation of the Wnt signaling pathway and may provide potential insights for the development of therapeutic targets.

## 1. Introduction

Stroke is an acute life-threatening condition in which poor blood perfusion in the brain causes cell death. Life style modification and pharmacological interventions to manage risk factors have been used to lower the incidence of strokes worldwide [1]. Despite such efforts, stroke is still associated with high mortality and morbidity across the globe [2]. Ischemic reperfusion injury in the brain is detrimental and elicits major dysfunctions within the body, such as impairments in movement, cognition and other vital functions [3]. Ischemic reperfusion injury in the brain encompasses abnormal production of oxygen radicals that may exacerbate initial ischemic injuries [4]. Such injury can occur in the current clinical therapies for stroke, such as thrombectomy. These surgeries are effective to ameliorate ischemic damage in the brain by the elimination of blockages [5]. However, it is currently arduous to prevent further oxidative damage caused by reperfusion. Again, secondary damage caused by reperfusion is intractable and could render leaving the reversible areas, such as the penumbra, vulnerable to oxidative damage [6]. Meanwhile, increases in inflammatory markers have been shown to reflect ischemic injuries in clinical settings. Cytokines, such as interleukin-1β (IL-1β), interleukin-6 (IL-6), interleukin-8 (IL-8), tumor necrosis factor-α (TNF-α), ficolin-1 and others, have been reported to increase in relations with the incident of stroke [7,8,9,10]. Therefore, decreasing inflammatory activity may be a goal for effective therapies. In previous studies, the administration of recombinant interleukin-1 receptor antagonist in rat models of middle cerebral artery occlusion (MCAO) successfully provided protection against ischemic injury [11,12]. However, the protective effects of interleukin-1 receptor antagonist have not been able to translate to neuroprotection in clinical trials but remain as a potential therapy through continued research [7].

Previous studies have implicated the importance of the canonical Wnt signaling pathway in the case of ischemic reperfusion injury [13,14]. The activation of glycogen synthase kinase-3β (GSK-3β) and the downregulation of protein kinase B (Akt) have been reported as important factors that contribute to the death of neurons [15,16]. The modulation of these proteins has been reported to affect survival of neurons in excitotoxic conditions [16,17]. The activity of GSK-3β leads to the degradation of downstream survival markers such as β-catenin through ubiquitination. Furthermore, canonical Wnt signals, which are lipid-modified glycoproteins, have been reported to inactivate GSK-3β through phosphorylation and thereby promote cell survival [18]. A recent study reported that motor exercise stimulated the canonical Wnt/β-catenin pathway for recovering from focal cerebral ischemic reperfusion injury in juvenile rats. Motor activity regulated canonical Wnt/β-catenin pathway and promoted neurogenesis and myelin repair [19]. Another study reported that electroacupuncture in multiple acupoints of a paralyzed limb stimulated the proliferation of neural progenitor cells through the activation of the Wnt/β-catenin signaling pathway and suppression of GSK-3β [20].

Lipid emulsion (LE) was approved for clinical use as a component of parenteral nutrition in 1962 [21]. LE is composed of 20% soybean oil, 1.2% egg yolk phospholipids, 2.25% glycerin and water for injection (Intralipid™ 20%, Fresenius Kabi, Uppsala, Sweden). The major fatty acids that constitute soybean oil are linoleic acid (44–62%), oleic acid (19–30%), palmitic acid (7–14%), linolenic acid (4–11%) and stearic acid (1.4–5.5%). In 1998, Weinberg et al. [22] shed light on the cardioprotective properties of LE against local anesthetic toxicity. Clinical reports have utilized the administration of LE for resuscitation from cardiac toxicity induced by lidocaine [23], ropivacaine [24] or bupivacaine [25]. The mechanism of protection of cardiac cells from excitotoxic conditions by LE depends on the phosphorylation of Akt [26] and GSK-3β [27] that contributes to cell survival. LE also has been reported to provide cardioprotection against ischemic reperfusion injury in the isolated rat heart [28]. While research into the utilization of LE against various cardiotoxic conditions is being actively conducted, investigations delineating the protective properties of LE in the central nervous system are still lacking. Neurons and cardiomyocytes share key similarities such as excitability and conductibility, which lead to their vulnerability to excitotoxic conditions. Relevant reports have claimed that distinct cardioprotective properties of LE might provide therapeutic targets or diagnostic tools for neuroprotection [29,30]. Our recent study has reported the protective effects of LE against kainic acid-induced excitotoxicity when administered directly into the brain, thereby revealing the potential neuroprotective aspects of LE [31]. However, the neuroprotective effects of LE against ischemic reperfusion injury in the brain have not been clearly elucidated.

Therefore, the present study investigated the neuroprotective effects of LE against ischemic reperfusion injury and elucidates the mechanism involved in the protection process. We examined the neuroprotective roles of LE in an in vivo rat model of the middle cerebral artery occlusion (MCAO) and reperfusion. We assessed the neurological deficits using the modified Bederson score [32] and extracted the brain to measure the severity of infarctions in experimental groups. We assessed the changes in protein and mRNA expression of distinct genes related to cell survival, Wnt/β-catenin signaling pathway and inflammation. We verified that XAV939, a Wnt/β-catenin signaling pathway inhibitor, reversed the protective effects. Based on our results, we propose that LE provides neuroprotection against ischemic reperfusion injury in the brain by regulating the Wnt/β-catenin signaling pathway.

## 2. Results

### 2.1. Dosage-Dependent Reduction in Infarction and Behavior by LE

**Figure 1:**
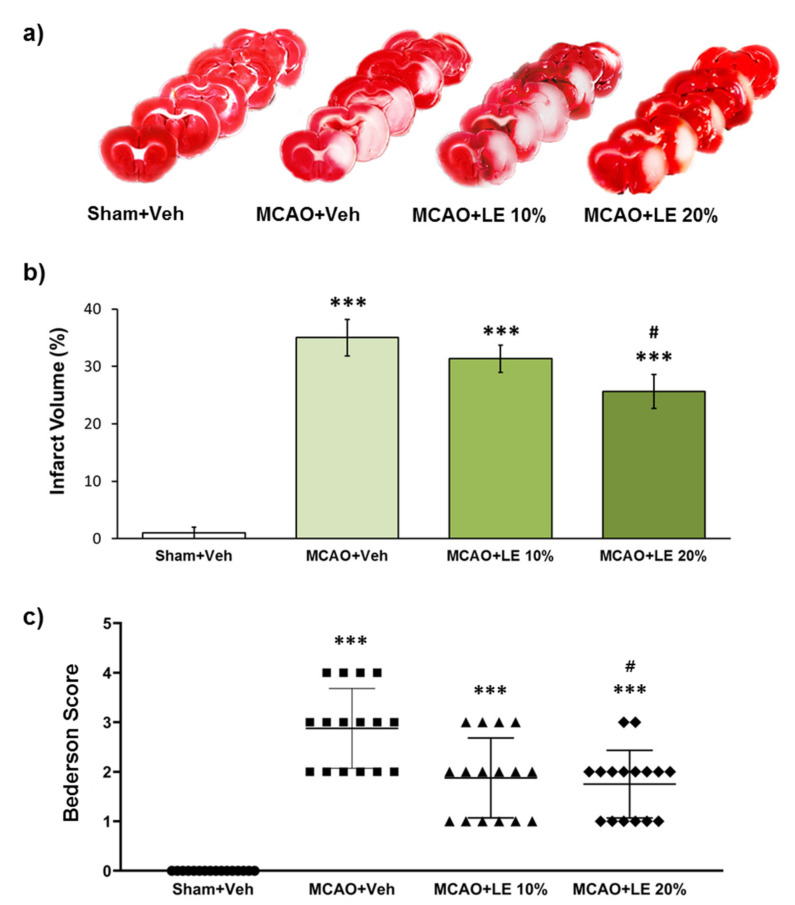
Neuroprotective effects of lipid emulsion (LE) or vehicle (Veh) after the middle cerebral artery occlusion (MCAO) and reperfusion injury. (**a**) Triphenyltetrazolium chloride (TTC)-stained brain slices for infarction measurement. Decrease in infarction volumes were observed in MCAO+LE 10% and MCAO+LE 20% groups. Sham group did not show ischemic reperfusion injury; (**b**) Measurement of infarction volume from TTC staining. The MCAO+Veh group increased significantly in infarction volume compared to Sham+Veh group. Both MCAO+LE 10% and MCAO+LE 20% decreased in infarction volume but only the MCAO+LE 20% group had significant difference compared to the MCAO+Veh group; (**c**) Bederson scores of the experimental groups. The MCAO+Veh group increased in Bederson score significantly compared to Sham+Veh group. Both MCAO+LE 10% and MCAO+LE 20% groups decreased in Bederson scores compared to MCAO+Veh group; however, only the MCAO+LE 20% group decreased significantly. Data are presented as mean ± standard error of the mean (SEM); *n* = 16 for each group; *** *p* < 0.001 vs. Sham+Veh, ^#^ *p* < 0.05 vs. MCAO+Veh. Statistical analysis for the measurement of infarction volume was performed using one-way analysis of variance (ANOVA) followed by Tukey’s multiple comparison test. Statistical analysis for Bederson scores was performed using Kruskal-Wallis non-parametric test followed by Dunn’s post hoc test.

The severity of infarction was measured by triphenyltetrazolium chloride (TTC) staining and neurological deficit assessment. The severity of infarction was visible through the unstained areas of the brain in Figure 1a. The Sham+Vehicle (Veh) group did not experience notable infarction. The MCAO+Veh group suffered an injury of approximately 35% of the left hemisphere. The MCAO+LE 10% group exhibited decrease in infarction volume to about 31%, which was not significant compared to MCAO+Veh group (*p* > 0.05, one-way ANOVA). There was a significant decrease in infarction to about 26% in the MCAO+LE 20% group compared to the MCAO+Veh group (*p* < 0.05, one-way ANOVA followed by Tukey’s multiple comparison test) (Figure 1b). In behavioral test, all experimental groups suffered a certain degree of behavioral deficit except for the Sham+Veh group. The MCAO+Veh group had an average Bederson score of 3. The MCAO+LE 10% group scored lower in the behavior test and did not differ significantly compared to the MCAO+Veh group (*p* > 0.05, Kruskal-Wallis non-parametric test). The administration of LE 20% significantly decreased the average Bederson score to approximately 2 (*p* < 0.05, Kruskal-Wallis non-parametric test followed by Dunn’s post hoc test). The majority of the MCAO+LE 20% group achieved a Bederson score of 2 or under, while the majority MCAO+Veh group recorded between 2 and 4. (Figure 1c).

### 2.2. Wnt/β-Catenin-Dependent Reduction in Infarction and Behavior by LE

**Figure 2:**
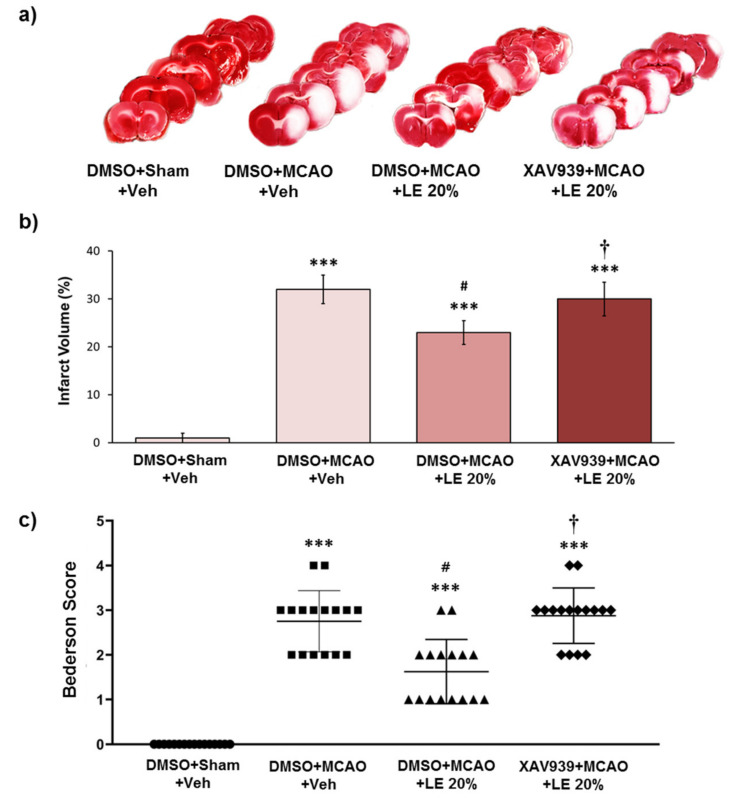
Neuroprotective effects of LE or vehicle on the MCAO and reperfusion injury after the administration of DMSO or XAV939. (**a**) TTC-stained brain slices for infarction measurement. Decrease in infarction volume was observed in the DMSO+MCAO+LE 20% group but not in the XAV939+MCAO+LE 20% group. Sham group (DMSO+Sham+Veh) did not experience ischemic reperfusion injury; (**b**) Measurement of infarction volume by TTC staining. The DMSO+MCAO+Veh group exhibited significant increase in infarction volume compared to the DMSO+Sham+Veh group. DMSO+MCAO+LE 20% group decreased significantly with respect to infarction volume when compared to the DMSO+MCAO+Veh. The XAV939+MCAO+LE 20% group had significantly increased infraction volume compared to the DMSO+MCAO+LE 20% group; (**c**) Bederson scores of experimental groups. The DMSO+MCAO+Veh group significantly increased in Bederson scores compared to the DMSO+Sham+Veh group. The DMSO+MCAO+LE 20% group showed significant decrease in Bederson scores compared to the DMSO+MCAO+Veh groups. The XAV939+MCAO+LE 20% group significantly increased in Bederscon scores compared to the DMSO+MCAO+LE 20% group. Data are presented as mean ± standard error of the mean (SEM); *n* = 16 for each group; *** *p* < 0.001 vs. DMSO+Sham+Veh, ^#^ *p* < 0.05 vs. DMSO+MCAO+Veh, ^†^ *p* < 0.05 vs. DMSO+MCAO+LE 20%. Statistical analysis for the measurement of infarction volume was performed using one-way ANOVA followed by Tukey’s multiple comparison test. Statistical analysis for Bederson scores was performed using Kruskal-Wallis non-parametric test followed by Dunn’s post hoc test.

Experimental groups were administered with intra-peritoneal (i.p.) injection of XAV939 to inhibit the Wnt/β-catenin signaling pathway induced by LE. The control group was administered with DMSO instead of XAV939. The severity of infarction was visible through the unstained areas of the brain as depicted in Figure 2a. The DMSO+Sham+Veh group did not experience notable infarction. Approximately 33% of the left hemisphere of the DMSO+MCAO+Veh group suffered an injury, while a significant decrease in infarction to about 24% was observed in the DMSO+MCAO+LE 20% group (*p* < 0.05, one-way ANOVA followed by Tukey’s multiple comparison test). The XAV939+MCAO+LE 20% failed to protect the brain with approximately 29% infarction volume, which was not significantly different from the DMSO+MCAO+Veh group (*p* > 0.05, one-way ANOVA followed by Tukey’s multiple comparison test) (Figure 2b). All experimental groups suffered a certain degree of infarction except for the DMSO+Sham+Veh group. The DMSO+Sham+Veh group did not experience notable neurological deficits. The DMSO+MCAO+Veh group recorded an average Bederson score of 3. The administration of LE 20% decreased the Bederson score to approximately 2 (*p* < 0.05, Kruskal-Wallis non-parametric test followed by Dunn’s post hoc test). The XAV939+MCAO+Veh group achieved an average Bederson score of 3, which was similar to the DMSO+MCAO+Veh group (*p* > 0.05, Kruskal-Wallis non-parametric test) (Figure 2c). The i.p. injection of DMSO did not induce significant differences in the experimental group.

### 2.3. LE Dosage-Dependent Alleviation of Ischemic Reperfusion Injury through the Wnt/β-Catenin Signaling Pathway and Reduction of Inflammatory Protein Markers

**Figure 3:**
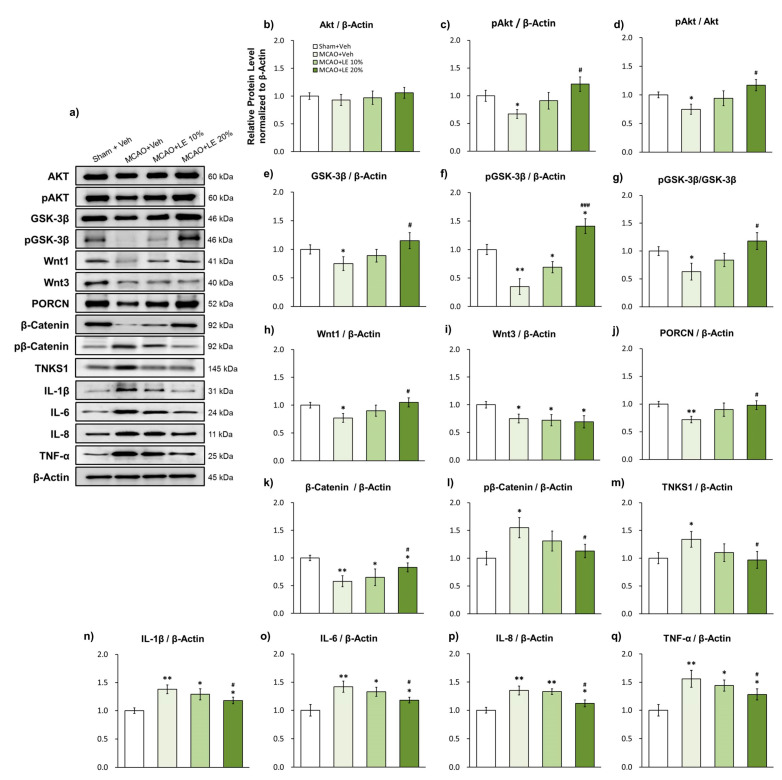
Effects of LE or vehicle on protein expressions after MCAO and reperfusion injury. (**a**) Representative Western blots indicating the expression of specific proteins in the penumbra region of the left hemisphere; (**b**–**d**) Expression and phosphorylation of Akt in the experimental groups. pAkt levels in MCAO+Veh group decreased significantly compared to the Sham+Veh group. pAkt was significantly increased in the MCAO+LE 20% group compared to the MCAO+Veh group; (**e**–**g**) Expression and phosphorylation levels of GSK-3β (pGSK-3β) in the experimental groups. pGSK-3β and GSK-3β levels in the MCAO+Veh group was significantly lower than the Sham+Veh group. The MCAO+LE 20% had significantly increased pGSK-3β and GSK-3β levels compared to the MCAO+Veh group; (**h**–**m**) Wnt signal-related protein expressions of experimental groups. Decreased levels of Wnt1, Wnt3, PORCN and β-catenin were observed in the MCAO+Veh group compared to the Sham+Veh group. The MCAO+LE 20% group had significantly increased protein levels of Wnt1, PORCN and β-catenin compared to MCAO+Veh group. Increased levels of pβ-catenin and tankyrase 1 were observed in the MCAO+Veh group compared to the Sham+Veh group. The MCAO+LE 20% group had significantly decreased levels of pβ-catenin and tankyrase 1 compared to the MCAO+Veh group; (**n**–**q**) Inflammatory protein expressions of experimental groups. Significantly increased expressions of inflammatory markers were observed in MCAO-injured groups compared to the Sham+Veh group. The MCAO+LE 20% group had significantly decreased inflammatory protein expression levels compared to the MCAO+Veh group. Data are presented as mean ± standard error of the mean (SEM); *n* = 8 for each group; * *p* < 0.05, ** *p* < 0.01 vs. Sham+Veh, ^#^ *p* < 0.05, ^###^ *p* < 0.001 vs. MCAO+Veh, one-way ANOVA followed by Tukey’s multiple comparison test.

The administration of LE affected the protein expression of survival and inflammation-related signals (Figure 3a). The expression of total Akt did not differ among the experimental groups (Figure 3b). The MCAO+Veh group showed significant decrease in phosphorylation of Akt levels (pAkt) compared to the Sham+Veh group (*p* < 0.05, one-way ANOVA followed by Tukey’s multiple comparison test). The MCAO+LE 10% group also exhibited an increase in pAkt levels. However, such an increase was not statistically significant compared to the MCAO+Veh group (*p* > 0.05, one-way ANOVA). The significantly elevated level of pAkt in MCAO+LE 20% group compared to the MCAO+Veh group indicated the increased survival of neurons after ischemic reperfusion injury (*p* < 0.05, one-way ANOVA followed by Tukey’s multiple comparison test) (Figure 3c). The ratio of pAkt and Akt was markedly decreased in the MCAO+Veh group compared to the Sham+Veh group (*p* < 0.05, one-way ANOVA followed by Tukey’s multiple comparison test). pAkt/Akt increased in expression in a dosage dependent manner in MCAO+LE 10% and MCAO+LE 20% groups; but only the MCAO+LE 20% group was significantly different from the MCAO+Veh group (*p* < 0.05, one-way ANOVA followed by Tukey’s multiple comparison test) (Figure 3d). 

Significant decrease in total GSK-3β expression was observed in the MCAO+Veh group compared to the Sham+Veh group (*p* < 0.05, one-way ANOVA followed by Tukey’s multiple comparison test). The MCAO+LE 10% group showed substantial increase in GSK-3β levels but this increment was not statistically significant compared to the MCAO+Veh group (*p* > 0.05, one-way ANOVA). The total GSK-3β expression of the MCAO+LE 20% was also significantly increased compared to the MCAO+Veh group (*p* < 0.05, one-way ANOVA followed by Tukey’s multiple comparison test) (Figure 3e). The phosphorylation of GSK-3β (pGSK-3β) was significantly decreased in the MCAO+Veh group compared to the Sham+Veh group (*p* < 0.01, one-way ANOVA), which might be indicating a diminished Wnt activity. The phosphorylation of GSK-3β was significantly increased in the MCAO+LE 10% (*p* < 0.05, one-way ANOVA followed by Tukey’s multiple comparison test) and MCAO+LE 20% (*p* < 0.001, one-way ANOVA followed by Tukey’s multiple comparison test) group when compared to the MCAO+Veh group. There was also a significant increase in pGSK-3β in the MCAO+LE 20% group compared to the Sham+Veh group (*p* < 0.05, one-way ANOVA followed by Tukey’s multiple comparison test), thereby indicating an increased activity of the Wnt/β-catenin signaling pathway (Figure 3f). The pGSK-3β/GSK-3β activity exhibited marked decrease in the MCAO+Veh group compared to the Sham+Veh group (*p* < 0.05, one-way ANOVA followed by Tukey’s multiple comparison test). The pGSK-3β/GSK-3β expression for MCAO+LE 10% did not differ significantly compared to the MCAO+Veh group (*p* > 0.05, one-way ANOVA). pGSK-3β/GSK-3β activity significantly increased in the MCAO+LE 20% group compared to the MCAO+Veh group (*p* < 0.05, one-way ANOVA followed by Tukey’s multiple comparison test) (Figure 3g). 

Wnt1, a canonical Wnt signal and upstream marker of GSK-3β, was significantly decreased in the MCAO+Veh group compared to the Sham+Veh group (*p* < 0.05, one-way ANOVA followed by Tukey’s multiple comparison test) and significantly increased in the MCAO+LE 20% group compared to the MCAO+Veh group (*p* < 0.05, one-way ANOVA followed by Tukey’s multiple comparison test) (Figure 3h). All experimental groups (*p* < 0.05, one-way ANOVA followed by Tukey’s multiple comparison test), excluding the Sham+Veh group, displayed robust decrease in the neurogenesis marker, Wnt3. Although Wnt3 is one of canonical Wnt signals, it does not seem to affect the Wnt/β-catenin signaling pathway induced by LE (Figure 3i). Porcupine (PORCN), a key regulator of Wnt proteins [33], decreased significantly in the MCAO+Veh group compared to the Sham+Veh group (*p* < 0.05, one-way ANOVA followed by Tukey’s multiple comparison test). PORCN expression increased in the MCAO+LE 20% group compared to the MCAO+Veh group, implying elevated activity in the modulation of Wnt proteins (Figure 3j). The downstream survival marker of GSK-3β, β-catenin, was decreased in all MCAO injury groups compared to the Sham+Veh group due to infarction. However, β-catenin was significantly preserved in the MCAO+LE 20% group compared to the MCAO+Veh group (*p* < 0.05, one-way ANOVA followed by Tukey’s multiple comparison test) (Figure 3k). Phosphorylation of β-catenin (pβ-catenin) was increased significantly in the MCAO+Veh group compared to the Sham+Veh group (*p* < 0.05, one-way ANOVA followed by Tukey’s multiple comparison test), indicating an elevated level of β-catenin degradation. The MCAO+LE 20% group had a significantly lower expression level of pβ-catenin compared to the MCAO+Veh group (*p* < 0.05, one-way ANOVA followed by Tukey’s multiple comparison test), which supported the survival of cells (Figure 3l). The expression level of tankyrase 1 was significantly increased in the MCAO+Veh group compared to the Sham+Veh group (*p* < 0.05, one-way ANOVA followed by Tukey’s multiple comparison test), indicating the accumulation of tankyrase 1 through increased axis inhibition protein (AXIN) stabilization for β-catenin degradation. The MCAO+LE 20% group had a significantly lower expression level of tankyrase 1 compared to the MCAO+Veh group (*p* < 0.05, one-way ANOVA followed by Tukey’s multiple comparison test) (Figure 3m). 

Inflammatory protein markers of ischemic reperfusion damage, IL-1β, IL-6, IL-8 and TNF-α [34,35], increased significantly for all MCAO-injured groups when compared to the Sham+Veh group. However, attenuated levels of inflammatory markers were observed in the MCAO+LE 20% group compared to the MCAO+Veh group (*p* < 0.05, one-way ANOVA followed by Tukey’s multiple comparison test), indicating the reduction of ischemic reperfusion injury (Figure 3n–q).

### 2.4. Wnt/β-Catenin-Dependent Alleviation of Ischemic Reperfusion Injury and Reversal of Protection-Related Proteins

**Figure 4:**
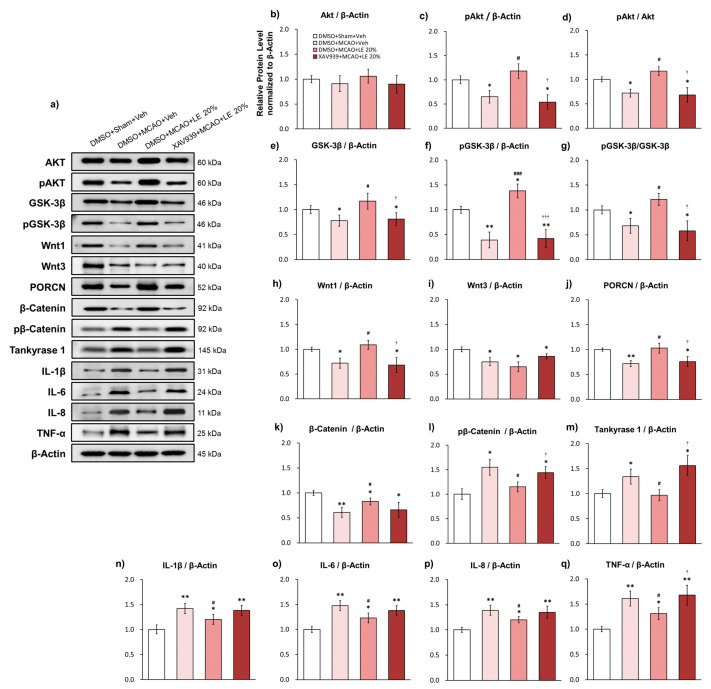
Effects of LE or vehicle on protein expression on the MCAO and reperfusion injury after the administration of DMSO or XAV939. (**a**) Representative Western blots of proteins in the penumbra region of the left hemisphere; (**b**–**d**) Levels of Akt and pAkt in the experimental groups. The DMSO+MCAO+Veh group and XAV939+MCAO+LE 20% group decreased significantly in pAkt level compared to DMSO+Sham+Veh group. pAkt was significantly increased in the DMSO+MCAO+LE 20% group compared to the DMSO+MCAO+Veh group and XAV939+MCAO+LE 20% group; (**e**–**g**) Levels of GSK-3β and pGSK-3β in the experimental groups. GSK-3β and pGSK-3β levels of DMSO+MCAO+Veh group and XAV939+MCAO+LE 20% group were significantly lower than the DMSO+Sham+Veh group. The DMSO+MCAO+LE 20% had significantly increased pGSK-3β and GSK-3β levels compared to the DMSO+MCAO+Veh and XAV939+MCAO+LE 20% groups; (**h**–**m**) Wnt signal-related protein expressions of experimental groups. Significantly decreased levels of Wnt1, Wnt3, PORCN and β-catenin were observed in the DMSO+MCAO+Veh group compared to the DMSO+Sham+Veh group. The DMSO+MCAO+LE 20% group had significantly increased protein levels of Wnt1 and PORCN compared to the DMSO+MCAO+Veh group. The XAV939+MCAO+LE 20% group had decreased expression levels of Wnt1 and PORCN compared to the DMSO+MCAO+LE 20% group. The β–catenin expression level in the DMSO+MCAO+LE 20% and XAV939+MCAO+LE 20% groups did not differ significantly. There was a significant increase in pβ–catenin and tankyrase 1 in the DMSO+MCAO+Veh compared to the DMSO+Sham+Veh group. Pβ–catenin and tankyrase1 was significantly decreased in the DMSO+MCAO+LE 20% compared to the DMSO+MCAO+Veh group. The XAV939+MCAO+LE 20% group had significantly increased pβ–catenin and tankyrase 1 expression levels compared to the DMSO+MCAO+LE 20% group; (**n**–**q**) Inflammatory protein expressions in the experimental groups. Significantly increased expressions of inflammatory markers were observed in the MCAO-injured groups compared to the DMSO+Sham+Veh group. The DMSO+MCAO+LE 20% group had significantly decreased inflammatory protein expression levels compared to the DMSO+MCAO+Veh group. Significant decrease in inflammatory protein expressions were observed in the XAV939+MCAO+LE 20% group compared to the DMSO+Sham+Veh group. TNF-α expression level was increased significantly in the XAV939+MCAO+LE 20% group compared to the DMSO+MCAO+LE 20% group. Data are presented as mean ± standard error of the mean (SEM); *n* = 8 for each group; * *p* < 0.05, ** *p* < 0.01 vs. DMSO+Sham+Veh, ^#^ *p* < 0.05, ^###^ *p* < 0.001 vs. DMSO+MCAO+Veh, ^†^ *p* < 0.05, ^†††^ *p* < 0.001 vs. DMSO+MCAO+LE 20%, one-way ANOVA followed by Tukey’s multiple comparison test.

The administration of XAV939 inhibited the activity of the Wnt/β-catenin signaling pathway, which was reflected in the protein expression (Figure 4a) of distinct genes. The expression of total Akt did not differ among the experimental groups (Figure 4b). The DMSO+MCAO+Veh group and XAV939+MCAO+LE 20% group significantly showed substantial decrease in pAkt levels compared to the DMSO+Sham+Veh group (*p* < 0.05, one-way ANOVA followed by Tukey’s multiple comparison test). There was significant elevation in the level of pAkt in DMSO+MCAO+LE 20% group compared to the DMSO+MCAO+Veh group (*p* < 0.05, one-way ANOVA followed by Tukey’s multiple comparison test) and XAV939+MCAO+LE 20% group (*p* < 0.01, one-way ANOVA followed by Tukey’s multiple comparison test) (Figure 4c). The pAkt/Akt expression was decreased significantly in the DMSO+MCAO+Veh and XAV939+MCAO+LE 20% groups compared to the DMSO+Sham+Veh group (*p* < 0.05, one-way ANOVA followed by Tukey’s multiple comparison test). The expression of pAkt/Akt in the DMSO+MCAO+LE 20% group was significantly increased compared to DMSO+MCAO+Veh and XAV939+MCAO+LE 20% groups (*p* < 0.05, one-way ANOVA followed by Tukey’s multiple comparison test) (Figure 4d). 

Significant decrease in total GSK-3β expression was observed in the DMSO+MCAO+Veh and XAV939+MCAO+LE 20% groups when compared to the DMSO+Sham+Veh group (*p* < 0.05, one-way ANOVA followed by Tukey’s multiple comparison test). Total GSK-3β expression of the DMSO+MCAO+LE 20% group was also significantly increased compared to the DMSO+MCAO+Veh and XAV939+MCAO+LE 20% groups (*p* < 0.05, one-way ANOVA followed by Tukey’s multiple comparison test) (Figure 4e). The level of pGSK-3β was significantly decreased in the DMSO+MCAO+Veh and XAV939+MCAO+LE 20% groups compared to the DMSO+Sham+Veh group (*p* < 0.01, one-way ANOVA followed by Tukey’s multiple comparison test), which might be indicative of a compromised Wnt activity. The phosphorylation of GSK-3β was significantly increased in the DMSO+MCAO+LE 20% group compared to the DMSO+Sham+Veh group (*p* < 0.05, one-way ANOVA followed by Tukey’s multiple comparison test). There was also a significant increase in the pGSK-3β levels in the DMSO+MCAO+LE 20% group compared to the DMSO+MCAO+Veh and XAV939+MCAO+LE 20% groups (*p* < 0.001, one-way ANOVA followed by Tukey’s multiple comparison test) (Figure 4f). The pGSK-3β/GSK-3β activity was significantly decreased in the DMSO+MCAO+Veh and XAV939+MCAO+LE 20% groups when compared to the DMSO+Sham+Veh group (*p* < 0.05, one-way ANOVA followed by Tukey’s multiple comparison test) and significantly increased in the DMSO+MCAO+LE 20% group compared to the DMSO+MCAO+Veh and XAV939+MCAO+LE 20% groups (*p* < 0.05, one-way ANOVA followed by Tukey’s multiple comparison test). The administration of XAV939 effectively decreased the phosphorylation GSK-3β induced by LE (Figure 4g).

Wnt1 was significantly decreased in the DMSO+MCAO+Veh and XAV939+MCAO+LE 20% groups compared to the DMSO+Sham+Veh group (*p* < 0.05, one-way ANOVA followed by Tukey’s multiple comparison test) and significantly increased in the DMSO+MCAO+LE 20% group compared to the DMSO+MCAO+Veh and XAV939+MCAO+LE 20% groups (*p* < 0.05, one-way ANOVA followed by Tukey’s multiple comparison test) (Figure 4h). All experimental groups, excluding the DMSO+Sham+Veh group, showed steep decrease (*p* < 0.05, one-way ANOVA followed by Tukey’s multiple comparison test) in Wnt3 (Figure 4i). PORCN decreased significantly in the DMSO+MCAO+Veh group (*p* < 0.01, one-way ANOVA followed by Tukey’s multiple comparison test) and XAV939+MCAO+LE 20% group (*p* < 0.05, one-way ANOVA followed by Tukey’s multiple comparison test) compared to the DMSO+Sham+Veh group. PORCN expression increased in expression in the DMSO+MCAO+LE 20% group compared to the DMSO+MCAO+Veh and XAV939+MCAO+LE 20% groups (*p* < 0.05, one-way ANOVA followed by Tukey’s multiple comparison test) (Figure 4j). β-catenin was decreased in all MCAO injury groups compared to the DMSO+Sham+Veh group due to infarction. Significantly attenuated level of β-catenin was also observed in the XAV939+MCAO+LE 20% group, while DMSO+MCAO+LE 20% group were preserved significantly compared to DMSO+MCAO+Veh group (*p* < 0.05, one-way ANOVA followed by Tukey’s multiple comparison test). Average β-catenin expression decreased in the XAV939+MCAO+LE 20% group compared to the DMSO+MCAO+LE 20% group but not to a significant level (*p* > 0.05 one-way ANOVA) (Figure 4k). pβ-Catenin was increased in the DMSO+MCAO+Veh group compared to the DMSO+Sham+Veh group (*p* < 0.05, one-way ANOVA followed by Tukey’s multiple comparison test), indicating lowered cellular survival. The level of pβ-catenin was significantly decreased in the DMSO+MCAO+LE 20% group compared to the DMSO+MCAO+Veh group (*p* < 0.05, one-way ANOVA followed by Tukey’s multiple comparison test). The pβ-catenin expression level was significantly increased in the XAV939+MCAO+LE 20% group compared to the DMSO+MCAO+LE 20% group (*p* < 0.05, one-way ANOVA followed by Tukey’s multiple comparison test) (Figure 4l). The accumulation of tankyrase 1 was significant in the DMSO+MCAO+Veh compared to DMSO+Sham+Veh (*p* < 0.05, one-way ANOVA followed by Tukey’s multiple comparison test). The tankyrase 1 expression of DMSO+MCAO+LE 20% group significantly decreased compared to the DMSO+MCAO+Veh group (*p* < 0.05, one-way ANOVA followed by Tukey’s multiple comparison test). The XAV939+MCAO+LE 20% group had significantly higher levels of tankyrase 1 expression levels compared to the DMSO+MCAO+LE 20% group (*p* < 0.05, one-way ANOVA followed by Tukey’s multiple comparison test). The administration of XAV939 successfully inhibited tankyrase 1 activity which increased degradation of β-catenin (Figure 4m). 

Inflammatory protein markers of ischemic reperfusion damage, IL-1β, IL-6, IL-8 and TNF-α, also significantly increased for all MCAO-injured groups compared to the DMSO+Sham+Veh group. Attenuated levels of inflammatory markers were observed in the DMSO+MCAO+LE 20% group compared to the DMSO+MCAO+Veh group (*p* < 0.05, one-way ANOVA followed by Tukey’s multiple comparison test). The expression levels of the XAV939+MCAO+LE 20% group were not significantly different from the DMSO+MCAO+Veh group (*p* > 0.05, one-way ANOVA) The TNF-α expression level of the XAV939+MCAO+LE 20% group increased significantly compared to the DMSO+MCAO+LE 20% group (*p* < 0.05, one-way ANOVA followed by Tukey’s multiple comparison test) (Figure 4n–q).

### 2.5. LE Dosage-Dependent mRNA Expression Against Ischemic Reperfusion Injury

**Figure 5:**
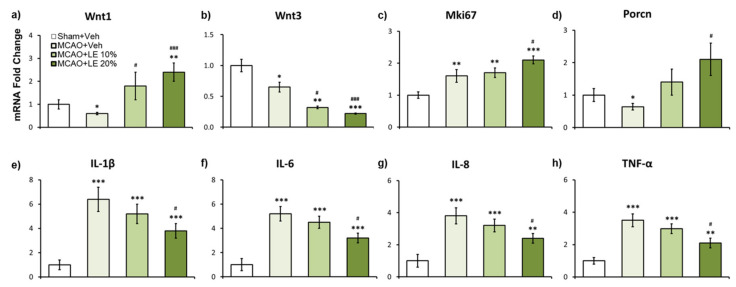
Effects of LE or vehicle on mRNA expression after the MCAO and reperfusion injury. (**a**,**b**) Wnt expressions in experimental groups. The Wnt1 mRNA expression of the MCAO+Veh group was significantly decreased compared to the Sham+Veh group. Significantly increased expression of Wnt1 was expressed in the MCAO+LE 10% and MCAO+LE 20% groups compared to the MCAO+Veh group. Wnt3 expressions were significantly lower in MCAO injury groups compared to the Sham+Veh group. Significantly decreased Wnt3 expressions were observed in the MCAO+LE 10% and MCAO+LE 20% groups compared to the MCAO+Veh group; (**c**) Mki67 expression was increased in all MCAO-injury groups compared to the Sham+Veh group. Significant increase in Mki67 expression was observed in the MCAO+LE 20% group compared to the MCAO+Veh group; (**d**) Porcn expression was significantly decreased in the MCAO+Veh group compared to the Sham+Veh group. Significant increase of Porcn was observed in the MCAO+LE 20% group compared to the MCAO+Veh group; (**e**–**h**) mRNA expression of inflammatory markers. MCAO-injury groups expressed significantly increased levels of inflammatory markers compared to the Sham+Veh group. Significantly decreased inflammatory expression levels were observed in the MCAO+LE 20% group compared to the MCAO+Veh group. Data are presented as mean ± standard error of the mean (SEM); *n* = 8 for each group; * *p* < 0.05, ** *p* < 0.01, *** *p* < 0.001 vs. Sham+Veh, ^#^ *p* < 0.05, ^###^ *p* < 0.001 vs. MCAO+Veh, one-way ANOVA followed by Tukey’s multiple comparison test. Wnt subfamily mRNA expressions are shown in Supplementary Materials S1.

According to the results of qPCR, Wnt1 can be implicated as one of the main regulators for neuroprotection. In the MCAO+Veh group, Wnt1 expression level was approximately 0.4 folds compared to the Sham+Veh group (*p* < 0.05, one-way ANOVA followed by Tukey’s multiple comparison test). The mRNA expression of Wnt1 signals of MCAO+LE 20% was upregulated by approximately 2.4 folds compared to the Sham+Veh group (*p* < 0.01, one-way ANOVA followed by Tukey’s multiple comparison test) and 4 folds compared to the MCAO+Veh group (*p* < 0.001, one-way ANOVA followed by Tukey’s multiple comparison test) (Figure 5a). Wnt3 expression was attenuated in all MCAO-injured groups compared to the Sham+Veh group. The MCAO+LE 10% (*p* < 0.05, one-way ANOVA followed by Tukey’s multiple comparison test) and MCAO+LE 20% groups’ (*p* < 0.001, one-way ANOVA followed by Tukey’s multiple comparison test) Wnt3 expression levels were significantly decreased compared to the MCAO+Veh group (Figure 5b). Mki67, a cell proliferation marker, increased in all MCAO-injured groups compared to the Sham+Veh group. The Mki67 expression level increased significantly in the MCAO+LE 20% group compared to the MCAO+Veh group (*p* < 0.05, one-way ANOVA followed by Tukey’s multiple comparison test), which may have been affected by the elevated expression level of Wnt1 (Figure 5c). The Wnt regulator, Porcn, decreased significantly in the MCAO+Veh group compared to the Sham+Veh group (*p* < 0.05, one-way ANOVA followed by Tukey’s multiple comparison test). Significant increase of Porcn was observed in the MCAO+LE 20% group compared to the MCAO+Veh group (*p* < 0.05, one-way ANOVA followed by Tukey’s multiple comparison test), which may be induced by the increase in Wnt1 activity (Figure 5d). Inflammatory markers, IL-1β, IL-6, IL-8 and TNF-α, significantly increased in MCAO-injured groups compared to the Sham+Veh group. Significantly lower inflammatory mRNA markers were observed in the MCAO+LE 20% group compared to the MCAO+Veh group (*p* < 0.05, one-way ANOVA followed by Tukey’s multiple comparison test) (Figure 5e–h).

### 2.6. Wnt-Dependent mRNA Expression of LE Against Ischemic Reperfusion Injury

**Figure 6:**
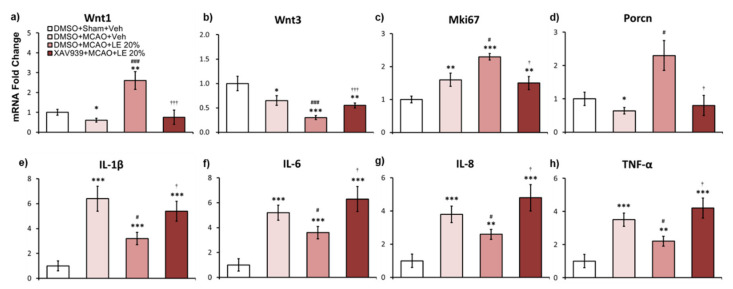
Effects of LE or vehicle on mRNA expression on the MCAO and reperfusion injury after the administration of DMSO or XAV939. (**a**,**b**) Wnt expressions in experimental groups. The Wnt1 mRNA expression of the DMSO+MCAO+Veh group was significantly decreased compared to the DMSO+Sham+Veh group. Significantly increased expression of Wnt1 was expressed in the DMSO+MCAO+LE 20% group compared to the DMSO+MCAO+Veh group. Wnt1 decreased significantly in the XAV939+MCAO+LE 20% group compared to the DMSO+MCAO+LE 20% group. There was no significant difference in Wnt1 expression in the XAV939+MCAO+LE 20% group compared to the DMSO+MCAO+Veh group. Wnt3 expressions were significantly lower in MCAO-injury groups compared to the DMSO+Sham+Veh group. Significantly decreased Wnt3 expressions were observed in the DMSO+MCAO+LE 20% group compared to the DMSO+MCAO+Veh and XAV939+MCAO+LE 20% groups; (**c**) Mki67 expression was increased in all MCAO-injury groups compared to the DMSO+Sham+Veh group. Significant increase in Mki67 expression was observed in the DMSO+MCAO+LE 20% group compared to the DMSO+MCAO+Veh. The XAV939+MCAO+LE 20% group had a significant decrease in Mki67 expression compared to the DMSO+MCAO+LE 20% group; (**d**) Porcn expression was significantly decreased in the DMSO+MCAO+Veh group compared to the DMSO+Sham+Veh group. Significant increase of Porcn was observed in the DMSO+MCAO+LE 20% group compared to the DMSO+MCAO+Veh. The XAV939+MCAO+LE 20% group had a significant decrease in Porcn expression compared to the DMSO+MCAO+LE 20% group. There was no significant difference in Porcn expression in the XAV939+MCAO+LE 20% group compared to the DMSO+MCAO+Veh group; (**e**–**h**) The mRNA expression of inflammatory markers. MCAO injury groups expressed significantly increased levels of inflammatory markers compared to the DMSO+Sham+Veh group. Significantly decreased inflammatory expression levels were observed in the DMSO+MCAO+LE 20% group compared to DMSO+MCAO+Veh group. Significantly increased levels of inflammatory markers were observed in the XAV939+MCAO+LE 20% group compared to the DMSO+MCAO+LE 20% group and DMSO+Sham+Veh group but there was no significant difference compared to the DMSO+MCAO+Veh group. Data are presented as mean ± standard error of mean (SEM); *n* = 8 for each group; * *p* < 0.05, ** *p* < 0.01, *** *p* < 0.001 vs. DMSO+Sham+Veh, ^#^ *p* < 0.05, ^###^ *p* < 0.001 vs. DMSO+MCAO+Veh, ^†^ *p* < 0.05, ^†††^ *p* < 0.001 vs. DMSO+MCAO+LE 20%, one-way ANOVA followed by Tukey’s multiple comparison test. Wnt subfamily mRNA expressions are shown in Supplementary Materials S1.

XAV939 injection inhibited Wnt activity in mRNA levels to reverse neuroprotection. In the DMSO+MCAO+Veh group (*p* < 0.05, one-way ANOVA followed by Tukey’s multiple comparison test) and XAV939+MCAO+LE 20% group, Wnt1 mRNA expression levels were approximately 0.5 folds and 0.6 folds compared to the DMSO+Sham+Veh group, respectively. Expression of Wnt1 signals in the DMSO+MCAO+LE 20% was upregulated by approximately 2.2 folds compared to the DMSO+Sham+Veh group (*p* < 0.01, one-way ANOVA followed by Tukey’s multiple comparison test) and 4.2 folds compared to the DMSO+MCAO+Veh group (*p* < 0.001, one-way ANOVA followed by Tukey’s multiple comparison test). There was a significant decrease in Wnt1 expression in the XAV939+MCAO+LE 20% group compared to the DMSO+MCAO+LE 20% group (*p* < 0.01, one-way ANOVA followed by Tukey’s multiple comparison test) (Figure 6a). Wnt3 expression was attenuated in all MCAO-injured groups compared to the DMSO+Sham+Veh group. The DMSO+MCAO+LE 20% group’s Wnt3 expression was significantly decreased compared to the DMSO+MCAO+Veh group (*p* < 0.001, one-way ANOVA followed by Tukey’s multiple comparison test). The XAV939+MCAO+LE 20% group was also showed significant decreased in Wnt3 expression level compared to the DMSO+Sham+Veh group (*p* < 0.01, one-way ANOVA followed by Tukey’s multiple comparison test) but displayed marked increase when compared to the DMSO+MCAO+LE 20% group (*p* < 0.001, one-way ANOVA followed by Tukey’s multiple comparison test) (Figure 6b). Mki67 levels increased in all MCAO-injured groups compared to the DMSO+Sham+Veh group. In the DMSO+MCAO+LE 20% group, Mki67 expression level increased significantly compared to the DMSO+MCAO+Veh group (*p* < 0.05, one-way ANOVA followed by Tukey’s multiple comparison test); while in the XAV939+MCAO+LE 20% group, it was significantly downregulated compared to the DMSO+MCAO+LE 20% group (*p* < 0.05, one-way ANOVA followed by Tukey’s multiple comparison test) (Figure 6c). Porcn decreased significantly in the DMSO+MCAO+Veh group (*p* < 0.05, one-way ANOVA followed by Tukey’s multiple comparison test) and XAV939+MCAO+LE 20% group compared to the DMSO+Sham+Veh group. Significant increase of Porcn was observed in the DMSO+MCAO+LE 20% group when compared to the DMSO+MCAO+Veh group (*p* < 0.05, one-way ANOVA followed by Tukey’s multiple comparison test). The XAV939+MCAO+LE 20% group decreased in Porcn compared to the DMSO+Sham+Veh group but did not differ significantly. The XAV939+MCAO+LE 20% group expression levels of Porcn were significantly decreased compared to the DMSO+MCAO+LE 20% group (*p* < 0.05, one-way ANOVA followed by Tukey’s multiple comparison test) (Figure 6d). Inflammatory markers, IL-1β, IL-6, IL-8 and TNF-α, significantly increased in MCAO-injured groups compared to the DMSO+Sham+Veh group. Significantly lower inflammatory mRNA markers were observed in the DMSO+MCAO+LE 20% group compared to the DMSO+MCAO+Veh group (*p* < 0.05, one-way ANOVA). XAV939 injection significantly reversed attenuated levels of inflammation by LE 20% (Figure 6e–h).

## 3. Discussion

Our results suggest that LE provides neuroprotection against ischemic reperfusion injury in a dosage-dependent manner. Intra-arterial injection of 20% LE during reperfusion after MCAO was able to reduce infarction significantly. The decreases in infarction volumes have been reflected in the improved performance in the neurological deficit assessment. Rats injected with LE 20% possessed better control over their paralyzed limb. Increased levels of Wnt1 signaling in both protein and mRNA levels in 20% LE-treated rats were observed, which in turn enhanced the resistance to reperfusion injury following ischemia. Especially, a notable increase in Wnt1 mRNA expression was observed, which may have led to increased Wnt1 protein expression. The elevated levels of Wnt1 are consistent with previous studies that have indirectly stimulated Wnt/β-catenin signaling pathway for cell survival after ischemic injury [20,36]. The expression levels of Wnt3 decreased in all MCAO groups which may be due to a decline in cell populations as a result of ischemic reperfusion injury [37]. Although Wnt3 is also known as a canonical Wnt signal, not all canonical Wnt signals seem to be involved in the protection process. As in our previous study [31], Wnt3 did not seem to be involved in protection process in response to the administration of LE. In addition, the phosphorylation of GSK-3β increased due to 20% LE injection, which inactivated the β-catenin destruction complex. β-catenin was preserved to promote cell survival, which resulted in enhanced resistance to the ischemic reperfusion damage. Elevation of PORCN expression may have been due to the increase in demand for Wnt1 to resist damage; however, a clear link between PORCN and LE cannot be formulated at this stage. A previous study on the ischemic reperfusion injury in the heart has reported that palmitic acid of LE affects the lipid-modification of Wnt ligands for protection [38]. Clear distinctions between cardiac cells and neurons exist but Wnt modifications seem to occur regardless of their metabolic factors. Inflammatory markers, IL-1β, IL-6, IL-8 and TNF-α, are known to increase after ischemic reperfusion injury [34,35]. Inflammation by oxidative stress was significantly decreased in the 20% LE-treated group, indicating an anti-inflammatory action of LE in the central nervous system. The preservation of β-catenin through the activation of the Wnt signaling pathway induces the transcription of T cell factor/lymphoid enhancer factor, which results in decreased inflammatory activity [39]. Therefore, the expressions of inflammatory cytokines, including IL-1β, IL-6 and TNF-α, might be reduced. The decrease in the expression of IL-8 is assumed to be reduced by an overall decline in inflammatory activity.

**Figure 7:**
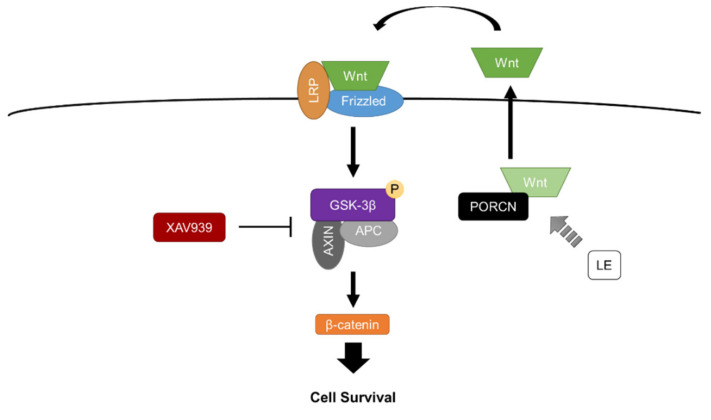
Schematic diagram of the protective mechanism of LE in ischemic reperfusion injury. LE increases the lipid modification of Wnt proteins by PORCN. Lipid-modified/active Wnt proteins are released to the extra-cellular space for the binding to Frizzled/LRP receptors. The activated receptors induce the phosphorylation of GSK-3β, which prevents the destruction complex from the degradation of β-catenin by proteasomes. Preserved levels of β-catenin lead to cell survival. The injection of XAV939 prevents the phosphorylation of GSK-3β, which allows β-catenin degradation for cell death. Abbreviations: PORCN—porcupine, Wnt—wingless integration, LRP—low-density lipoprotein receptor-related protein, GSK-3β—glycogen synthase kinase 3β, APC—adenomatous polyposis coli, AXIN—axis inhibition protein.

The Wnt/β-catenin signaling pathway has been considered a potential therapeutic target in preventing cell death for an extensive amount of time [18,40,41]. The down-regulation of Wnt ligands and increased antagonistic activity have been observed in neurodegenerative and excitotoxic disorders [42,43,44]. In order to verify the protective mechanism of LE, which increased Wnt1, we were required to interrupt the protective process. XAV939, a Wnt/β-catenin signaling pathway inhibitor, has been commonly utilized to inhibit Wnt activity in many studies [45,46]. In the present study, the i.p. injection of XAV939 successfully inhibited Wnt activity and reversed the protective effects induced by LE. The XAV939+MCAO+LE 20% group exhibited infarction volumes nearly as large as our control, the DMSO+MCAO+Veh group. Akin to earlier studies, our results show that the Wnt/β-catenin signaling pathway might be a potential therapeutic target for neuroprotection. The administration of XAV939 inhibited the mRNA expression level of Wnt1, indicating that Wnt1 may be dependent on the activity of GSK-3β. XAV939 is known to inhibit tankyrase 1, which prevents the degradation of axis inhibition protein 2 (AXIN2), allowing the accumulation of the GSK-3β destruction complex [47]. Therefore, pGSK-3β was attenuated in the XAV939+MCAO+LE 20% group, which reversed the protective effect of LE by the degradation of the downstream survival marker, β-catenin (Figure 4k). We can infer that LE has effects on the upstream signals of GSK-3β and not on the downstream signals of GSK-3β (Figure 7). If LE affected the GSK-3β downstream marker, β-catenin, XAV939 might not have affected the protection effect. In addition, if LE affected GSK-3β directly, a less effective or partial reversal of protection may have occurred. We observed that the infarction damage was not significantly different between the DMSO+MCAO+Veh group and XAV939+MCAO+LE 20% group, implying an effective reversal from the effects of 20% LE injection.

The protective roles of fatty acids have received much attention in studies regarding stroke. Different compositions of lipid emulsions, such as omega-3, have been reported to provide neuroprotection against ischemic stroke injuries [48]. Emulsions of n-3 fatty acids have been shown to effectively reduce infarction volume and inflammation in the ischemic brain in neonatal mice [49]. In the present study, we observed significant neuroprotective effects of LE against ischemic reperfusion injury but limitations exist. Although we were able to investigate protective properties of LE regarding the Wnt/β-catenin signaling pathway, we are yet to discover specific targets of LE. Further studies should consider other signals that are involved in the Wnt/β-catenin signaling pathway, such as frizzled-1, low-density lipoprotein receptor-related protein 5/6 or PORCN, that can be subjected for inhibition. Elucidation of specific signals may lead to more effective therapeutic targets in the brain. Through our study, we suggested that the neuroprotective mechanism of LE is dependent on the Wnt/β-catenin signaling pathway through the inhibition of GSK-3β phosphorylation. We assumed that lipid modification occurs through PORCN through the constituents of LE that interact with the protein. As a result, the expression levels of Wnt1 increased, which also affects its downstream signals. The scope for future studies is considerable: LE may be utilized with other lipophilic anti-inflammatory drugs to further extend its neuroprotective properties. Furthermore, the investigation of different routes of delivery may also be considered with regard to invasiveness and safety.

In conclusion, the intra-arterial administration of 20% LE alleviated ischemic reperfusion injury induced by MCAO and reperfusion. Infarction volumes and Bederson scores were attenuated in a dosage-dependent manner. Protein and mRNA expression levels of the Wnt/β-catenin signaling pathway were elevated and inflammatory markers decreased significantly in the 20% LE-treated group. Especially, GSK-3β was phosphorylated significantly, which in turn preserved β-catenin to promote cell survival. The protective actions were reversed by the administration of XAV939 indicating that the protection mechanism of LE has been induced through the Wnt/β-catenin signaling pathway. These findings regarding the anti-inflammatory and neuroprotective properties of LE may provide a foundation for further research regarding excitotoxic neural injuries and functional recovery.

## 4. Materials and Methods

### 4.1. Animals

Adult Sprague-Dawley rats weighing 260-300 g (Koatec, Pyeongtaek, South Korea) were used for experiments in this study. Animals were housed in groups of three per cage under 12-h light/dark cycles, with free access to food and water. Animals were subjected to 7 days of acclimatization upon arrival at the Association for Assessment and Accreditation of Laboratory Animal Care (AAALAC)-accredited Yonsei University College of Medicine Animal Care Facilities. All experimental procedures were approved by the Institutional Animal Care and Use Committee of Yonsei University Health System (permit no.: 2019-0263, approval date: 22 January 2020) and performed according to the National Institutes of Health Guide for Care.

### 4.2. Middle Cerebral Artery Occlusion and Reperfusion

**Figure 8:**
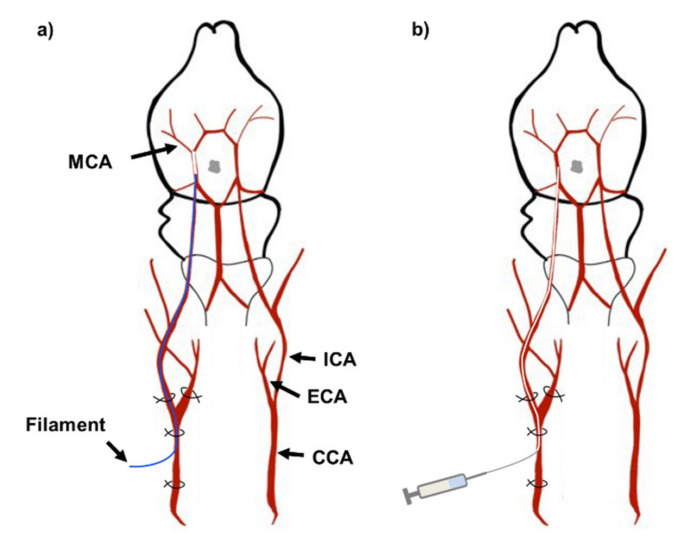
Illustration of middle cerebral artery occlusion and injection. (**a**) Insertion of nylon filament for MCAO. The CCA was ligated permanently while the ECA and ICA were temporarily ligated to stop blood flow. A small incision was made in the CCA for the filament insertion. The silicone-coated filament was inserted until it reached the MCA. The artery was occluded for 90 min. Then the filament was removed for reperfusion; (**b**) Insertion of polyethylene tubing for injection of LE or vehicle. The polyethylene tubing was inserted near to the MCA. After injection, the tubing was removed and the CCA near the bifurcation was permanently ligated. The temporary ligations on the ICA and ECA were removed. Abbreviations: CCA—common carotid artery, ECA—external carotid artery, ICA—internal carotid artery, MCA—middle cerebral artery.

Rats were anesthetized by i.p. injection of 50 mg/kg sodium pentobarbital (Hanlim Pharmaceutical, Seoul, South Korea) and were placed on a heated mat to maintain body temperature of 37.0 ± 1.0 °C. The surgical and injection process is illustrated in Figure 8. The Koizumi method [50] of MCAO was used for inducing ischemic reperfusion injury. Cervical skin incision was made to expose the common carotid artery (CCA), external carotid artery (ECA) and internal carotid artery (ICA). The CCA was permanently ligated below the bifurcation to the ECA and ICA. The ECA was temporarily ligated while a small incision was made in the CCA to insert a silicone coated nylon filament (403723PK10Re, Doccol Corporation, Sharon, MA, USA) into the ICA until a mild resistance stopped the insertion. The length of the insertion was approximately 18-20 mm (Figure 8a). The rat was occluded for 90 min. and the filament was removed. The experimental group undergoing sham surgery did not have the filament inserted. Polyethylene tubing with an inner diameter of 0.28 mm and outer diameter of 0.61 mm (427401, Becton, Dickson and Company, Sparks, MD, USA) was inserted for injection of LE or vehicle (Figure 8b). Injections were made at 0.5 mL/min over 2 min. The polyethylene tubing was removed after the injection and the CCA above the incision was permanently ligated. The temporary ligation of the ECA was removed. A total of 374 rats were used for this study. The MCAO surgery was conducted on 310 rats and sham surgery was conducted on 64 rats. 84 rats that deceased during MCAO, reperfusion or intra-arterial injection were excluded from the experiment. None of Sham-operated rats deceased. 34 rats that underwent MCAO and reperfusion but did not show infarction due to surgical errors were also excluded from the study.

### 4.3. Drug Treatment

LE (Intralipid™ 20%, Fresenius Kabi, Uppsala, Sweden) was dissolved in sterile 0.9% NaCl to a final concentration of 10% for the injection of the LE 10% group. Vehicle, 10% LE and 20% LE were intra-arterially administered during reperfusion after 90 min. of MCAO. Intra-peritoneal injection XAV939 (S1180, Selleck Chemicals, Houston, TX, USA) 40mg/kg or DMSO were injected for 2 days prior to the MCAO surgery and on the day of the surgery before MCAO.

### 4.4. Neurological Deficit Assessment

A neurological examination of experimental animals was performed in accordance with the modified Bederson method [51] 24 h after MCAO or sham surgery by a blinded researcher. The scoring criteria are as follows: score 0: no deficit; score 1: lost forelimb flexion; score 2: as for 1, plus decreased resistance to lateral push; score 3: unidirectional circling; score 4: longitudinal spinning or seizure activity; score 5: no movement.

### 4.5. Infarction Volume Assessment

The infarction volume was assessed by TTC staining of consecutive 2.0 mm coronal sections from bregma +4 mm to −6.0 mm. The sections were immersed into 2% TTC (T8877, Sigma Aldrich, St. Louis, MO, USA) solution for 30 min at 37.0 °C. The sections were transferred into 4% paraformaldehyde solution for 24 h. The sections were then photographed and analyzed using the ImageJ software (ImageJ, National Institute of Health, Bethesda, MD, USA). The infarction volume was calculated using the following formula [50]: (contralateral volume – non-infarct ipsilateral volume)/contralateral volume × 100%. The contralateral volume refers to the opposite brain hemisphere of the infarcted side.

### 4.6. Western Blotting

Frozen samples were homogenized for protein extraction in lysis buffer (ProPrep; Intron Biotechnology, Pyeongtaek, South Korea) with phosphatase inhibitors (Phosstop; Roche, Mannheim, Germany). Supernatants were collected from homogenized samples that were centrifuged at 15,000 rpm for 15 min at 4 °C. Total protein concentrations were measured using a spectrophotometer (Nano Drop ND-1000, NanoDrop Technologies Inc., Wilmington, DE, USA) and equal amount of protein (30 mg per well) were resolved on 10% sodium dodecyl sulfate-polyacrylamide gel electrophoresis (SDS-PAGE) and transferred to a polyvinylidene difluoride membrane (Merck Millipore, Darmstadt, Germany) for 2 h. Phospho-proteins were detected by immunoblotting prior to their corresponding total protein levels, which were detected after the membrane had been stripped. Transferred proteins on membranes were fixed using 0.05% glutaraldehyde in Tris-buffered saline containing 0.05% Tween-20 (TBST) for 15 min at room temperature. The membrane was stained with Ponceau S (P7170, Sigma-Aldrich, St. Louis, MO, USA) for visualization of the transferred proteins and cut into strips according to the size of the target protein to minimize interactions between antibodies. Membranes were blocked with 5% bovine serum albumin (BSA) dissolved in TBST for 1 h at room temperature. The membranes were incubated overnight with primary antibodies diluted in 5% BSA in TBST at 4 °C. Rabbit was the host of all primary antibodies used for immunoblotting. The following antibodies were used—anti-Wnt1 (ab15251, 1:1000, Abcam, Cambridge, UK), anti-Wnt3 (ab32249, Abcam), anti-PORCN (ab105543, Abcam), anti-β-catenin (#9562, 1:3000, Cell Signaling Technology, Beverly, MA, USA), anti-Akt (#4691, 1:3000, Cell Signaling Technology), anti-Phospho-Akt (#9271, 1:1000, Cell Signaling Technology), anti-GSK3-β (#9315, 1:3000, Cell Signaling Technology), anti-Phospho-GSK3-β (#9336, 1:1000, Cell Signaling Technology), anti-pβ-catenin (#9561, 1:1000, Cell Signaling Technology), anti-tankyrase 1 (MBS8531631, 1:1000, MyBioSource, San Diego, CA, USA), anti-IL-1β (AB1832P, 1:1000, Sigma Aldrich), anti-IL-6 (ARC0062, 1:500, Invitrogen, Carlsbad, CA, USA), anti-IL-8 (MBS9385550, 1:500, MyBioSource), anti-TNF-α (AAR33, 1:1000, Bio-Rad, Hercules, CA, USA) and anti-β-actin (#4967, 1:10,000, Cell Signaling Technology). After overnight incubation of primary antibodies, the membranes were incubated with anti-rabbit horseradish peroxidase-conjugated secondary antibody (#7074, 1:10,000, Cell Signaling Technology). Visualization of immunoreactive proteins was performed with the application of chemiluminescent detection reagent (ECL™ Prime, GE Healthcare, Little Chalfont, UK) and images were taken by ImageQuant™ LAS 4000 (GE Healthcare). Protein immunoreactivity was measured using Multi-gauge software (Fuji Film Inc., Tokyo, Japan).

### 4.7. qPCR

RNA was extracted by using the Hybrid-R kit (305-010; GeneAll Biotechnology, Seoul, Korea). The concentration of RNA was measured using a spectrophotometer (Nano Drop ND-1000, NanoDrop Technologies Inc). cDNA was prepared from 1 μg of total RNA using the PrimeScript 1st strand cDNA synthesis kit (Takara Bio, Shiga, Japan). PCR amplification was executed using the SYBR-Green reagent (Takara Bio) in the ABI 7500 real-time PCR system (Applied Biosystems, Foster City, CA, USA). PCR amplification was performed in 20 μL reaction volumes. Sequences for oligonucleotide primers were selected using the Gene Database of National Center for Biotechnology Information (NCBI) and Primer Express™ Software v3.0.1 (Thermo Fisher Scientific, Waltham, MA, USA). Primer pairs are listed in Table 1.

### 4.8. Statistical Analysis

Statistical evaluations were performed using one-way ANOVA or unpaired *t*-test, as indicated in figure legends. Post hoc analyses were performed using the Tukey’s multiple comparisons test or as otherwise specified in the figure legends. All statistical analyses were performed using GraphPad Prism software (GraphPad Software Inc., San Diego, CA, USA). A *p*-value less than 0.05 was considered statistically significant for all analyses.

## Figures and Tables

**Table 1:** Primer pairs for qPCR.

Gene Name	Forward Primer (5′-3′)	Reverse Primer (5′-3′)
*Wnt1*	GCAACCAAAGTCGCCAGAA	TATGTTCACGATGCCCCACCA
*Wnt2b*	GCTACCCAGACATCATGCG	ACACTCTCGGATCCATTCCC
*Wnt3*	AATTTGGTGGTCCCTGGC	GATAGAGCCGCAGAGCAGAG
*Wnt4*	GTTTCCAGTGGTCAGGATGC	AGGACTGTGAGAAGGCTACGC
*Wnt5a*	AAGGGAACGAATCCACGCC	ATACTGTCCTGCGACCTGCTTC
*Wnt7a*	CCAAGGTCTTCGTGGATGC	TGTAAGTTCATGAGGGTTCGG
*Wnt7b*	CGTGTTTCTCTGCTTTGGC	CACCACGGATGACAATGC
*Wnt9a*	GTACAGCAGCAAGTTTGTCAAGG	CACGAGGTTGTTGTGGAAGTCC
*Wnt10a*	CGGAACAAAGTCCCCTACG	AGGCGAAAGCACTCTCTCG
*Wnt16*	GCACTCTGTAACCAGGTCATGC	TGCAAGGTGGTGTCACAGG
Mki67	TTCAGTTCCGCCAATCCAAC	CCGTGCTGGTTCCTTTCCA
*P* *orcn*	CCTACCTCTTCCCCTACTTCA	CTTTGCGTTTCTTGTTGCGA
*IL-1β*	AATGCCTCGTGCTGTCTG	TCCATTGAGGTGGAGAGC
*IL-6*	ATGAAGTTTCTCTCCGCAAG	CAACAACATCAGTCCCAAG
*IL-8*	AGCCTTCCTGATTTCTGC	AGCACTCCTTGGCAAAAC
*TNFα*	CAGCCGATTTGCCATTTC	TCTTGATGGCAGAGAGGAG
*β-Actin*	GTCCACCCGCGAGTACAAC	TATCGTCATCCATGGCGAACTGG

## Supplementary Materials

Supplementary Materials can be found at https://www.mdpi.com/1422-0067/21/19/7373/s1.

## Abbreviations

AAALAC	Association and Accreditation of Laboratory Animal Care
ANOVA	analysis of variance
APC	adenomatous polyposis coli
AXIN	axis inhibition protein
CCA	common carotid artery
DMSO	dimethyl sulfoxide
ECA	external carotid artery
GSK3-β	glycogen synthase kinase-3β
ICA	internal carotid artery
IL	interleukin
LE	lipid emulsion
LRP	low-density lipoprotein receptor-related protein
MCA	middle cerebral artery
MCAO	middle cerebral artery occlusion
mRNA	messenger ribonucleic acid
PORCN	porcupine
qPCR	quantitative polymerase chain reaction
SEM	standard error of mean
TNF-α	tumor necrosis factor-α
Veh	vehicle
Wnt	wingless integration
XAV939	2-[4-(trifluoromethyl)phenyl]-7,8-dihydro-5H-thiino [4,3-d]pyrimidin-4-ol
