# Supplementary material for: Lipid Emulsion Improves Functional Recovery in an Animal Model of Stroke
Source: Int J Mol Sci. 2020 Oct 6;21(19):7373. doi: 10.3390/ijms21197373 (PMC7582956; doi:10.3390/ijms21197373)
Supplement: Supplementary file 1 [file ijms-21-07373-s001.pdf]

International Journal of *Molecular Sciences*

MDPI

Supplementary Materials

Lipid emulsion improves functional recovery in an animal model of stroke

Motomasa Tanioka [1,2], Wyun Kon Park [3], Joohyun Park [2,4], Jong Eun Lee [2,4], and Bae Hwan Lee [1,2,*]

[1] Department of Physiology, Yonsei University College of Medicine, Seoul 03722, Republic of Korea; hpark@yuhs.ac (M.T.)
[2] Brain Korea 21 PLUS Project for Medical Science, Yonsei University College of Medicine, Seoul 03722, Republic of Korea
[3] Department of Anesthesiology and Pain Medicine, Anesthesia and Pain Research Institute, Yonsei University College of Medicine, Seoul 03722, Republic of Korea; wkp7ark@yuhs.ac (W.K.P.)
[4] Department of Anatomy, Yonsei University College of Medicine, Seoul 03722, Republic of Korea; jhpark922@yuhs.ac (J.P.); jelee@yuhs.ac (J.E.L.)
* Correspondence: bhlee@yuhs.ac; Tel.: +82-2-2228-1711

a)

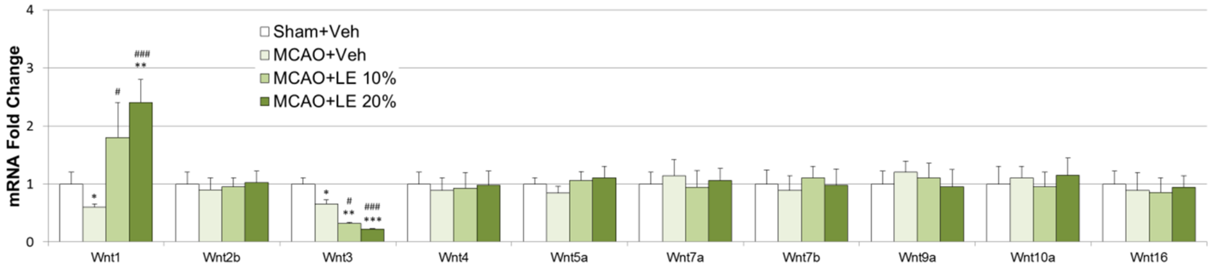

b)

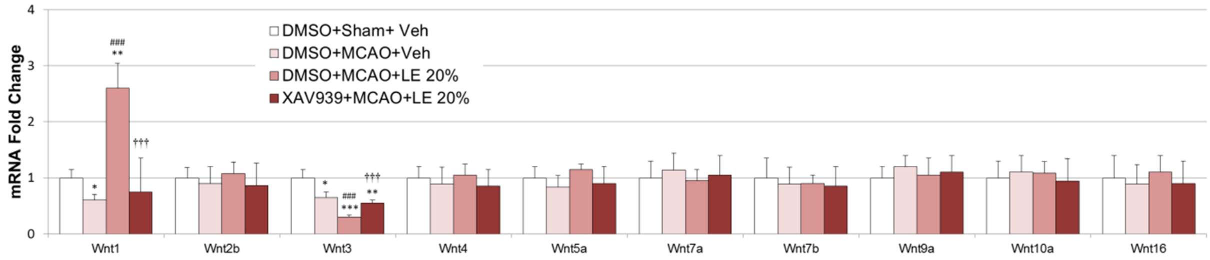

Supplementary Material S1. mRNA expression level of *Wnt* subfamilies. (**a**) LE dosage-dependent mRNA expression level of Wnt subfamilies. The *Wnt1* mRNA expression of the MCAO+Veh group was significantly decreased compared to the Sham+Veh group. Significantly increased expression of *Wnt1* was expressed in the MCAO+LE 10% and MCAO+LE 20% groups compared to the MCAO+Veh group. *Wnt3* expressions were significantly lower in MCAO injury groups compared to the Sham+Veh group. Significantly decreased *Wnt3* expressions were observed in the MCAO+LE 10% and MCAO+LE 20% groups compared to the MCAO+Veh group. There was no significant difference in other *Wnt* subfamilies. Data are presented as mean ± standard error of mean (SEM); n=8 for each group; *P<0.05, **P<0.01, ***P<0.001 vs Sham+Veh, #P<0.05, ###P<0.001 vs. MCAO+Veh, one-way analysis of variance (ANOVA) followed by Tukey's multiple comparison test. (**b**) mRNA expression level of DMSO or XAV939 treated experimental groups. The *Wnt1* mRNA expression of the DMSO+MCAO+Veh group was significantly decreased compared to the DMSO+Sham+Veh group. Significantly increased expression of *Wnt1* was expressed in the DMSO+MCAO+LE 20% group compared to the DMSO+MCAO+Veh group. There was no significant difference in *Wnt1* expression in the XAV939+MCAO+LE 20% group compared to the DMSO+MCAO+Veh group. *Wnt1* decreased significantly in the XAV939+MCAO+LE 20% group compared to the DMSO+MCAO+LE 20% group. *Wnt3* expressions were significantly lower in MCAO-injury groups compared to the DMSO+Sham+Veh group. Significantly decreased *Wnt3* expressions were observed in the DMSO+MCAO+LE 20% group compared to the DMSO+MCAO+Veh and XAV939+MCAO+LE 20% groups. There was no significant difference in other *Wnt* subfamilies. Data are presented as mean ± standard error of mean (SEM); n=8 for each group; *P<0.05, **P<0.01, ***P<0.001 vs DMSO+Sham+Veh, ###P<0.001 vs. DMSO+MCAO+Veh, †††P<0.001 vs. DMSO+MCAO+LE 20%, one-way analysis of variance (ANOVA) followed by Tukey's multiple comparison test.

© 2020 by the authors. Submitted for possible open access publication under the terms and conditions of the Creative Commons Attribution (CC BY) license (http://creativecommons.org/licenses/by/4.0/).
